# Scaling up Semi-Arid Grassland Biochemical Content from the Leaf to the Canopy Level: Challenges and Opportunities

**DOI:** 10.3390/s101211072

**Published:** 2010-12-06

**Authors:** Yuhong He, Amy Mui

**Affiliations:** Department of Geography, University of Toronto Mississauga, 3359 Mississauga Road North, Mississauga, Ontario, L5L 1C6, Canada; E-Mail: amy.mui@utoronto.ca

**Keywords:** semi-arid grasslands, vegetation biochemical content, hyperspectral remote sensing, scaling, leaf level, canopy level

## Abstract

Remote sensing imagery is being used intensively to estimate the biochemical content of vegetation (e.g., chlorophyll, nitrogen, and lignin) at the leaf level. As a result of our need for vegetation biochemical information and our increasing ability to obtain canopy spectral data, a few techniques have been explored to scale leaf-level biochemical content to the canopy level for forests and crops. However, due to the contribution of non-green materials (*i.e.*, standing dead litter, rock, and bare soil) from canopy spectra in semi-arid grasslands, it is difficult to obtain information about grassland biochemical content from remote sensing data at the canopy level. This paper summarizes available methods used to scale biochemical information from the leaf level to the canopy level and groups these methods into three categories: direct extrapolation, canopy-integrated approach, and inversion of physical models. As for semi-arid heterogeneous grasslands, we conclude that all methods are useful, but none are ideal. It is recommended that future research should explore a systematic upscaling framework which combines spatial pattern analysis, canopy-integrated approach, and modeling methods to retrieve vegetation biochemical content at the canopy level.

## 1. Introduction

One fifth of the Earth's land surface is covered by grasslands, both natural and human-altered. Semi-arid or temperate grasslands are found across the globe in every continent except Antarctica and are characterized by low levels of annual precipitation and distinct wet and dry seasons. Semi-arid grasses typically grow in clump-like formations due to the lack of moisture available to support full ground coverage. After each growing season, the above ground biomass remains on the surface of the soil until the next wet season arrives, when it is quickly converted into humus and incorporated into the dark and nutrient-rich soil that is characteristic of grasslands. It is this fertile soil that first drew the attention of agriculturalists, and has led to the North American prairies and the Russian steppes being named the ‘bread baskets’ of the world. Common to all grassland ecosystems across the globe is their importance to ecological, socio-economic, and agricultural activities.

Grassland degradation however, has become a worldwide problem due to intense human activities and environmental change. Degradation leads to complex physiological processes that change both the biochemical and biophysical properties of grassland vegetation. To accurately assess the health of remaining grasslands *in situ*, vegetation parameters need to be surveyed at different temporal and spatial scales. Some key grassland biophysical parameters (e.g., biomass and leaf area index) have been estimated from remotely sensed data [1,2]. However, biochemical parameters (e.g., chlorophyll, nitrogen, and lignin) are not always available in a spatially-explicit format due to the lack of appropriate data with high spectral and spatial resolutions across broad areas.

Biochemical parameters control physiological processes such as nutrient cycling, net primary production, photosynthetic capability and litter decomposition. Therefore knowledge of vegetation biochemical content is a key issue when describing, understanding and predicting an ecosystem’s health. For example, chlorophyll content is an indicator of leaf photosynthetic activity, which is directly related to the phenology and health status of plants [3]. Chlorophyll can be used to measure vegetation stress, life stage, productivity, and CO_2_ sequestration while the contents of lignin and nitrogen govern litter degradation. Further, the ability to specify spatially-explicit biochemical properties such as these, is especially important for semi-arid grasslands where the non-green vegetation component (e.g., litter), dominates the total fraction of aboveground biomass [4]. Non-photosynthetic grassland litter forms an important function by contributing a short-term pool of material and energy which flows from plant to soil, and varies with different microclimatic conditions.

Remote sensing of biochemical content at the leaf scale has been studied for several decades [5–6]. Many empirical and physical methods have been developed and successfully used for the determination of biochemical content in individual leaves. Empirical estimation of leaf-level biochemical content uses univariate or multivariate models to find a relation between the target biochemical parameter and spectral reflectance or some spectral indices. Most spectral indices employ ratios of narrow bands from spectral ranges that are sensitive to biochemicals, to those not sensitive. The purpose of spectral indices was to minimize variability caused by external factors such as illumination and atmosphere conditions. Examples of spectral indices developed for the study of vegetation biochemicals based on leaf reflectance can be found in recent published papers [7–11]. Reviews of different spectral indices developed for estimating chlorophyll, a particularly important biochemical, are offered by Haboudane *et al.* [8].

Physical methods involve radiative transfer (RT) models to estimate biochemical content at the leaf level [12–15]. The physical models must be inverted when retrieving vegetation characteristics from observed reflectance data [16]. The inversion of leaf-level radiative transfer models, such as PROSPECT and LEAFMOD, has been used to predict leaf biochemical content in forests and croplands [17–21].

To obtain spatially explicit grassland biochemical content, it is necessary to scale leaf-level biochemical measurements to the canopy level. However, canopy level vegetation reflectance is heavily influenced by vegetation type, its state, spatial distribution and canopy composition [22]. Therefore scaling up to the canopy level for the purposes of estimating biochemical content involves a very complex process with multiple inputs and methodologies, each of which can strongly alter results.

The recent availability of airborne and space-borne hyperspectral data has enabled new methods for estimating the biochemical properties of vegetation from the leaf to the canopy scale. Examples of airborne and spaceborne systems include CASI (Compact Airborne Spectrographic Imager) and MERIS (Medium Resolution Imaging Spectrometer). Using airborne and space-borne hyperspectral data, considerable efforts have been made to scale various vegetation parameters from the leaf to the canopy level in forests and crops [23–24].

Although research on vegetation biochemical estimations using hyperspectral remote sensing data has been commonly explored during the past decades, a comprehensive review of semi-arid grassland biochemical estimations at the canopy level is not yet available. This paper reviewed recent scaling techniques and discussed the major questions: (1) why is remote sensing of semi-arid grassland biochemicals unique, (2) what are the commonly-used methods for scaling up leaf-level biochemical to the canopy level based on hyperspectral remote sensing data; and (3) can we apply these methods directly to semi-arid grasslands and what are the challenges and opportunities for hyperspectral remote sensing of biochemicals in semi-arid grasslands?

## 2. Why Hyperspectral Remote Sensing of Semi-Arid Grassland Biochemicals Is Unique

The interaction of electromagnetic radiation with plant leaves is determined by their chemical and physical properties [25–26]. Vegetation biochemical absorption regions occur at more than forty specific wavelengths between 430 and 2,350 nm [27]. Remote sensing of vegetation biochemicals is an exploration of the chemical absorption regions of the electromagnetic spectrum based on the assessment of vegetation harvested and examined in laboratory settings.

**Figure 1.**
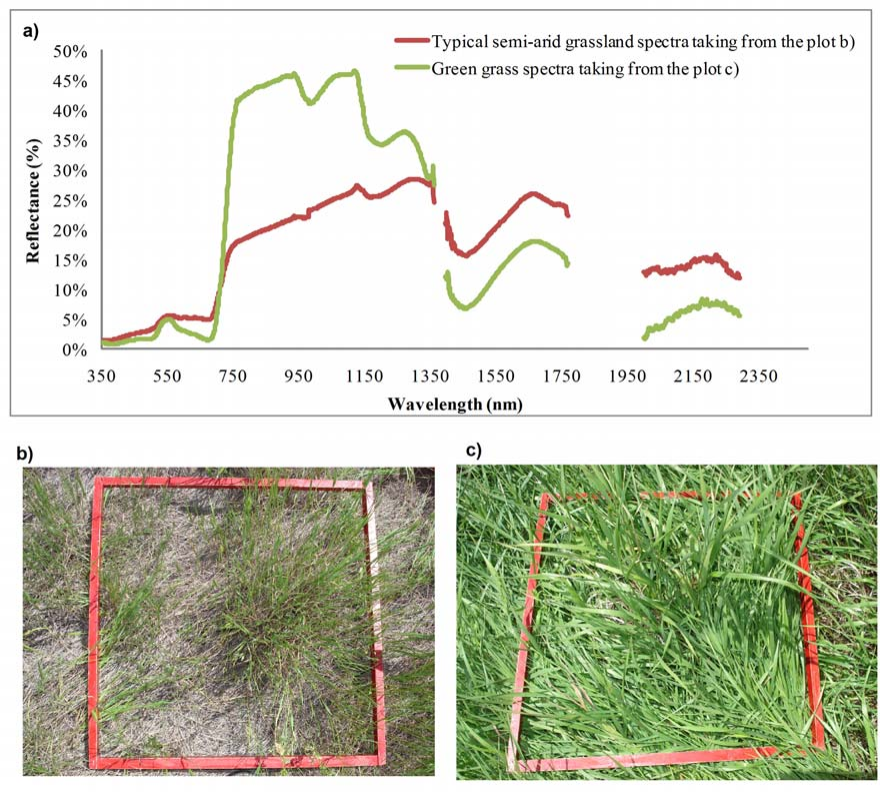
Hyperspectral response curves (**a**) of a semi-arid grassland and a green grass site. Three primary atmospheric water absorption (noisy) regions (1,361–1,395 nm, 1,811–1,925 nm, 2,350–2,500 nm) for the field measurements were deleted [1,28]. Photographs (**b**) and (**c**) were taken from the plots where the spectral reflectances were collected.

However, the sampling of spectra from a grassland canopy to assess biochemical properties necessarily encounters many challenges, including contributions from non-photosynthetic materials, atmospheric influences, and selection of appropriate methods of analysis. Figure 1(a) shows a typical semi-arid grassland canopy reflectance spectra from a semi-arid grassland site [Figure 1(b)] and typical green vegetation reflectance spectra collected from a site with green grass [Figure 1(c)]. Spectral reflectance of the semi-arid grassland had general features similar to that of typical vegetation in the red absorption region, near-infrared (NIR) reflectance region, and three atmospheric water absorption regions [1,28]. The absorption and reflectance in red and NIR regions, however, were not as strong as those of typical vegetation. For example, the reflectance collected from the semi-arid grasslands was higher in the red wavelength region and much weaker in the NIR region, compared to the spectral curve of typical green grass.

The primary spectral differences between semi-arid grassland vegetation and typical green vegetation are due to the contribution of non-green materials (*i.e.*, standing dead, litter, rock, and bare soil) to the canopy spectral response. Specifically, the effects of dead litter, which often dominates the total fraction of aboveground biomass [29] and varies with different microclimatic conditions [30], present a serious problem to the interpretation of remote sensing data [31]. The contribution of bare soil and soil brightness is also a significant barrier to the determination of vegetation biochemical properties [32–34]. It has been widely recognized that separating components of photosynthetic vegetation, non-photosynthetic vegetation and exposed soil in hyperspectral imagery are significant research challenges [35] and semi-arid grasslands are no exception.

## 3. Current Methods and Their Challenges for Scaling Vegetation Biochemical Content from the Leaf to the Canopy Level Based on Remote Sensing Data

### 3.1. Direct Extrapolation Method

The extrapolation method is the simplest way to scale remote sensing biochemicals from the leaf to the canopy level. This empirically-based method applies the leaf-level relationships between reflectance or reflectance indices and biochemical content directly to the canopy-level reflectance spectral data measured in the field or by airborne or satellite sensors [6,36,37].

The basic assumptions of the extrapolation method are that all leaves in the plant have the same biochemical content and only a fine layer of leaves covers an entire pixel in hyperspectral imagery. In vegetation with an almost complete cover, as is the case with some agricultural crops, a significant correlation between satellite signal must exist with the total leaf biochemical content in the leaves. However, for semi-arid grasslands, the leaf-level relationship may not be directly extrapolated to the canopy level across broad spatial and temporal scales as the complicated perturbations of canopy and vegetation composition to light transfer are not considered during the extrapolation. Several studies [15,36,38] have also demonstrated poor signal propagation from the leaf to the canopy scale in other heterogeneous systems.

### 3.2. Canopy-Integrated Method

To overcome the scaling issue related to the extrapolation method, the canopy-integrated biochemical content has been correlated to reflectance or reflectance indices. The canopy-integrated biochemical content can be obtained by multiplying the leaf biochemical content by the corresponding canopy biophysical parameters such as leaf area index or biomass. For example, in a grassland study conducted by Jago *et al.* [39], the chlorophyll content was defined as chlorophyll concentration × biomass within an area covered by a pixel. Similarly, Gitelson *et al.* [11] estimated total chlorophyll in maize canopies using LAI × leaf chlorophyll content. This canopy-integrated approach markedly improved current techniques proposed for biochemical quantification in the canopy. However, the major assumption of the canopy-integrated method is that all leaves in the plant have the same biochemical content. Consequently, the method might be successful when only one type of plant homogeneously covers each pixel of the hyperspectral image.

To scale leaf-level spectral-biochemical relationships to the canopy level for semi-arid grasslands, it might not be ideal to use the canopy-integrated method [40] for two reasons: (1) biochemical content is not uniformly distributed in all grassland species, and (2) optical remote sensing systems are very sensitive to non-green components of the canopy. For the latter reason, the fraction of non-green material (e.g., standing litter) must be accounted for in studies trying to retrieve biochemical content from optical reflectance at the canopy level. To address these issues, a new canopy-integrated approach and new spectral indices were developed. The new canopy-integrated approach considered that leaves in the canopy have different biochemical content, and therefore calculated the canopy biochemical content as the sum of the biochemical content of individual leaves of each canopy normalized to ground area. Meanwhile, the new spectral indices take species heterogeneity and non-green canopy components into account. To retrieve the canopy-level biochemical content, the statistical relationships between the calculated canopy biochemical content and spectral indices from new approaches were then established. An example of these new approaches can be found from Gitelson *et al.* [11]

The robustness and generality of the newly developed indices were tested over a range of species and canopy conditions in cropland. For example, a new index [(R_NIR_/R_λ_)-1] was proposed by Gitelson *et al.* [11] to address the species-specific issue. Using this index, canopy chlorophyll was found to be accurately estimated by current space-borne sensors such as MODIS and MERIS for maize and soybean crops with very different canopy architectures and leaf structures. However, the index was found to be species-specific in the spectral ranges of current space-borne sensors, thus different calibration coefficients may be required for different vegetation types, and estimation errors may increase under a mixed pixel scenario, which is common in semi-arid grasslands.

Another new index (Normalized Area Over reflectance Curve, NAOC) was developed for calculating vegetation chlorophyll content in heterogeneous areas with different species, different canopies and different types of bare soil [3]. By correlating ground- and space-level NAOC with chlorophyll experimental data for a broad range of crops, authors found the NAOC improved the accuracy of vegetation biochemical estimations. However, this method was only tested using data from cropland where other non-green components do not exist. Future studies are required to expand this method to semi-arid grasslands.

In summary, the canopy-integrated approach is an efficient means of scaling vegetation biochemical content from the leaf to the canopy level for homogenous croplands. The newly improved canopy-integrated approach may be applied to semi-arid grasslands as it adds up the biochemical content of individual leaves from the entire canopy. However, it is a time consuming approach as the biochemical content of individual leaves from the canopy has to be examined. The recently published spectral indices could be used for different species, different canopies and different types of bare soil. However, these indices do not take standing litter into consideration. The importance of standing litter on canopy reflectance has been demonstrated by Asner’s study [29] in which standing litter was found to have a disproportionately strong effect on grassland canopy reflectance, and changes in standing litter play a much stronger role in driving canopy reflectance variability than a concomitant change in any other structural attribute such as LAI.

### 3.3. Physical Models

Both the direct extrapolation method and the canopy-integrated method relied on statistical analyses to estimate individual biochemical variables of interest. However, the effects of canopy characteristics (structure and composition) on vegetation biochemical retrievals cannot be quantified using this statistical approach. Canopy biochemical content may vary with different canopy structural properties (*i.e.*, leaf area index and biomass), increasing the sensitivity of spectral data to canopy attributes [23]. Consequently, researchers turned to radiative transfer models as they can provide quantitative information about the covariance or decoupling of canopy effects in hyperspectral data [41]. The radiative transfer modeling simulates the radiation transfer processes in the canopy by computing the interaction between plants and solar radiation. Specifically, biochemical characteristics of vegetation at the canopy level are retrieved through the inversion of the canopy radiative transfer models [42–46]. In comparison with the direct extrapolation method and the canopy-integrated approach, these inversion models offer the potential of a more generic approach to quantifying vegetation biochemicals from hyperspectral data.

Different radiative transfer models have been proposed for different canopy characteristics ranging from homogenous canopy [15,47] to heterogeneous canopy [48–49]. The model developed for homogenous canopy presumed the vegetation canopy to be homogenous in composition, horizontal in shape and continuous [50]. The examples of this type of model are SAIL [15] and KUUSK [51]. Moorthy *et al.* [52] investigated the chlorophyll concentration of coniferous forests at the canopy level through a turbid medium canopy model named SAILH. Unfortunately, the turbid medium assumption used in this model did not account for heterogeneities in the canopies. Therefore, if applying this model to semi-arid grasslands, the turbid medium hypotheses would be violated, and further the model would not realistically simulate the canopy reflectance, thereby creating bias in the retrieved biochemical variables [14].

For situations in which the assumption of a horizontally homogeneous and continuous canopy does not apply, geometrical and three-dimensional models have been developed for horizontally heterogeneous or discontinuous canopies. Geometrical models can describe radiation propagation in sparse canopies where multiple scattering can be ignored and mutual shading is negligible due to low zenith angles [53]. Examples of three-dimensional models include TRIM [54], GeoSAIL [55], the Discrete Anisotropic Radiative Transfer (DART) model [48], and the INvertible FOrest Reflectance Model (INFORM) [56]. More recently, two more advanced radiative models - Monte Carlo ray tracing models [57,58] and radiosity models [59–61] give a more realistic representation of the radiation transfer in the canopy. Unfortunately, these models are very computationally intensive and have been rarely compared to each other in terms of accuracy or speed. Further, these models have been developed primarily for forest or crop canopy, and their suitability for semi-arid grasslands remains unknown.

Physical models have to be inverted in order to retrieve vegetation characteristics from observed reflectance data [62]. Commonly-used inversion algorithms include numerical optimization methods [14,63–65], look-up table (LUT) approaches [66–69], artificial neural network (ANN) methods [70–73], and support vector machines regression [74]. Each inversion technique has its own advantages and disadvantages for which a detailed discussion on this regard can be found in Kimes *et al.* [16] and Liang [54]. Here, we would like to point out that a major drawback of the inversion process is that the inverse solution is not always unique since various combinations of canopy parameters can yield almost similar spectra [79]. This problem is also called the ill-posed nature of model inversion [75].

Significant efforts to estimate and quantify vegetation biochemical properties using radiative transfer models have been carried out in the last two decades for agricultural crops [41,65,76–78], and for forests [79–85]. Despite numerous examples of successful application, a review of the literature reveals that radiative transfer models and inversion approaches have seldom been applied to estimate vegetation biochemical variables for heterogeneous semi-arid grasslands. Recently, Darvishzadeh *et al.* [1] explored the capability of the PROSAIL model and LUT inversion technique for estimating canopy chlorophyll contents in a heterogeneous Mediterranean grassland. The authors found that inverting a radiative transfer model using hyperspectral measurements to determine grass canopy chlorophyll content can be estimated with accuracies similar to those of empirical approaches. However, authors also indicated that the PROSAIL model is not well adapted to multi-species canopies, particularly under the turbid medium assumption, and the inversion of PROSAIL under such conditions leads to a bias in the retrieved biophysical parameters. The higher estimation accuracy may possibly be obtained through a three-dimensional model (because of its more realistic description of the reflected radiation field) or more advanced models.

## 4. Future Directions for Scaling Grassland Biochemical Content from the Leaf to the Canopy Level

Among the three methods discussed, the direct extrapolation method holds the least potential for estimating grassland biochemical variables at the canopy level because the physical assumptions used for the horizontal layers of leaves [86] are not applicable in semi-arid grasslands and would yield unrealistic solutions. Therefore, this method will not be discussed in the following section.

### 4.1. Opportunities Associated with the Canopy-Integrated Approach

The above mentioned gap opens up new opportunities for semi-arid grassland research. A promising research direction would focus on the improvement of current spectral indices which would be able to estimate canopy biochemical content, while reducing the effect of non-green components, especially standing dead litter. An example of an improved index can be the incorporation of a litter adjustment factor with a promising index such as NAOC as explained in He *et al.* [2]. As a result, the proposed improved NAOC would consider all components in semi-arid grasslands, including different species, canopy structure, soil types, and standing dead litter. Other research opportunities involve: (1) testing newly improved canopy-integrated approaches to obtain ground canopy biochemical content for semi-arid grasslands; (2) exploring the capability of published spectral indices and new spectral indices at the canopy scale using ground hyperspectral data, at the local scale using airborne high-spatial resolution (e.g., CASI 2- or 4-m) spectral data, and at the regional scale using spaceborne medium-spatial resolution (CHRIS/PROBA 36 m) data; (3) establishing a generic relationship between ground canopy biochemical content and airborne/spaceborne vegetation indices at different scales; and (4) validating the relationships in (3) based on multi-year field and space data.

### 4.2. Opportunities Associated with the Modeling Method

The few physically-based models that have been applied to grasslands reported difficulties with the inversion process, and indicated a need for input parameters at finer scales in order to accurately describe grassland canopy characteristics [1,87]. Currently, no studies have accurately estimated the biochemical content of vegetation in a grassland ecosystem. Accurately estimating grassland biochmical variables from the inversion of radiative modeling depends on: (1) the accuracy of acquired spectral data, (2) the suitability of the radiative model, (3) a priori knowledge of canopy characteristics to parameterize the inversion, and (4) the appropriate inversion process [88].

In response to the accuracy of acquiring spectral data for biochemical studies, major efforts have been made to reprocess existing data sets into forms more suitable for scientific use. Therefore, it will not be discussed in this section.

Previous discussion has indicated that the most suitable radiative transfer model for semi-arid grasslands could be three dimensional models or an advanced model designed for heterogeneous systems. However, their suitability remains to be examined. Future research should evaluate the performance of different three-dimensional or advanced models and potential inversion procedures with the focus on applying them operationally in the study of semi-arid grassland biochemical estimation. Further, the inversion of three-dimensional radiative models requires a high number of input parameters, and possible solutions to the ill-posed inverse problem involve the use of prior knowledge about model parameters [67] and the use of information provided by key canopy parameters at multiple temporal scales [89]. One major direction of future study is thus to obtain the input parameters at multiple-temporal scales [89] that are needed to parameterize the physical models. The input parameters include: (1) the type of canopy architecture, (2) the canopy composition, and (3) the typical distribution of canopy biochemical variables [1]. Combal *et al.* [66] as well as Meroni *et al.* [14] have shown that utilizing prior information of model inputs is an efficient way of solving the ill-posed problem and of improving the accuracy of the estimated canopy variables. The input parameters may be acquired from field measurements, GIS datasets, and other sources such as an expert experience.

### 4.3. Establishing a Systematic Upscaling Framework

Opportunities discussed above are limited to remote sensing data and methods. We should keep in mind that remote sensing imagery and methods are not the only approach that should be considered when dealing with upscaling issue for a heterogeneous ecosystem. Other approaches such as spatial pattern analysis should also be taken into account when spatial heterogeneity of land surface cannot be ignored. Spatial pattern analysis could be used to: (1) identify the scale of the heterogeneity (patchiness) of the landscape so that subsequent analyses will be conducted at an appropriate scale [90], and (2) produce pattern indices that used as independent variables in models attempting to establish a link between spatial pattern and ecological process. In the context of scaling grassland biochemical content from the leaf level to the canopy level, future research could focus on developing a systematic upscaling framework which integrates spatial pattern analysis with the canopy-integrated and modeling approach.

The systematic upscaling framework should consider following three steps: (1) performing spatial patch analysis to determine the dominant scale of the heterogeneity so that an appropriate remote sensing image can be selected for a specific semi-arid grassland [90]; (2) improving the canopy-integrated approach by using both the patch indices and remote sensing data/indices as independent variables to predict the canopy-level biochemicals. Therefore, the established empirical models not only consider all components in the canopy of semi-arid grasslands (different species, canopy structure, soil types, and standing dead litter), but also take the patchiness into account; (3) using the empirical relationships between canopy biochemical variables and patch and spectral indices developed in (2) to parameterize the radiative models. The spectral indices can be obtained from the use of hyperspectral remote sensing data with different angular, spectral, spatial, or temporal properties [91], and from the fusion of these hyperspectral data with LiDAR, thermal and microwave remote sensing. It is recommended that the information at multiple temporal and spatial scales may greatly help in choosing the initial parameter values for model inversion and may probably improve the regularization of the model inversion, thus overcoming the ill-posed problem [75]. Consequently, a more accurate estimation of the biochemical variables for semi-arid grasslands can be expected from such an integrated approach.

## 5. Conclusions

The ability to assess vegetation biochemical and biophysical properties at multiple spatial scales will lead to a better understanding of grassland conditions, especially under climate and environmental changes. Some key grassland biophysical properties (e.g., biomass and leaf area index) have been estimated from remote sensing data [1,91]. However, the other set of important properties, biochemicals, are not always available in a spatially-explicit format due to a lack of appropriate data at the required resolutions across a broad area. The recent availability of space-borne hyperspectral data has enabled new methods for estimating the biochemical properties of vegetation from the leaf to the canopy level. Studies have demonstrated that hyperspectral remote sensing data are useful for assessing vegetation biochemicals in continuous green canopies (crops and forests) at the canopy level. We summarized currently available techniques and concluded that three general classes of techniques have been applied to obtain vegetation biochemical properties at the canopy level: (1) the extrapolation method that applies leaf-level empirical relationships directly to the canopy level; (2) the canopy-integrated method that correlates biochemical measurements to spectral data/indices; and (3) the inversion of the physical model approach that attempts to offer the potential of a more generic method of quantifying vegetation biochemical content from hyperspectral data.

However, more research is needed to address the potential of remote sensing data for assessing biochemicals at the canopy level in low vegetated ecosystems like semi-arid grasslands, because semi-arid grasslands are unique to remote sensing studies in terms of its spectral properties. Through discussing the assumptions of each existing method, we concluded that, for semi-arid grasslands, the leaf-level relationship cannot be used through direct extrapolation to the canopy level across broad spatial and temporal scales because the complex canopy characteristics are not considered. In terms of the canopy-integrated approach and newly developed spectral indices (e.g., NAOC), further testing is recommended in order to examine the accuracy of estimating canopy biochemical content for semi-arid grasslands at multiple spatial and temporal scales. An improved index may be developed by incorporating a litter adjustment factor with an existing index such as NAOC so that all canopy components in semi-arid grasslands, including different species, canopy structure, soil types, standing dead and litter, will be considered. In terms of the physical modeling approach, research is needed to evaluate the performance of different three-dimensional or advanced models and potential inversion procedures with the focus on applying them operationally in the studies of semi-grassland biochemical estimations.

Other than the remote sensing data-based canopy-integrated approach and modeling method, future research should also consider spatial patch analyses because spatial heterogeneity of land surface in semi-arid grasslands cannot be ignored. Consequently, we recommend that future research should focus on developing a systematic upscaling framework which integrates spatial pattern analysis, the canopy-integrated approach, and the physical modeling method towards scaling grassland biochemicals from the leaf to the canopy level. The recommended systematic upscaling framework will be an efficient, reliable and repeatable methodology that could measure semi-arid grassland biochemical properties spatially. The resultant spatially distributed grassland biochemical maps will aid in the understanding of the relationships that exist between grasslands, disturbances, climate change, and community stability. For example, the biochemical maps will provide spatial estimates of forage/range quality that would be of great use ecologically for both macro & micro-grazer studies. In addition, the grassland biochemical maps will allow stakeholders (including park managers, ranchers, and policy makers) to make better decisions concerning conservation planning, stocking capacity estimation, grassland carbon accounting, and native grassland and rangeland management.

## References

[1] He Y, Guo X, Wilmshurst J (2006a). Studying mixed grassland ecosystems I: Suitable hyperspectral vegetation indices. Can. J. Remote Sens.

[2] Price KP, Guo X, Stiles JM (2002). Optimal Landsat TM band combinations and vegetation indices for discrimination of six grassland types in eastern Kansas. Int J Remote Sens.

[3] Delegido J, Alonso L, Gonza G, Moreno J (2010). Estimating chlorophyll content of crops from hyperspectral data using a normalized area over reflectance curve (NAOC). Int J Appl Earth Observation Geoinf.

[4] Deutsch ES, Bork EW, Willms WD (2010). Separation of grassland litter and ecosite influences on seasonal soil moisture and plant growth dynamics. Plant Ecol.

[5] Card DH, Peterson DL, Matson PA, Aber JD (1988). Prediction of leaf chemistry by the use of visible and near infrared reflectance spectroscopy. Remote Sens Environ.

[6] Peterson DL, Aber JD, Matson PA, Card DO, Swanberg N, Wessman C, Spanner M (1988). Remote sensing of forest canopy and leaf biochemical contents. Remote Sens Environ.

[7] Daughtry CC, Walthall L, Kim MS, De Colstoun EB, McMurtrey JE (2000). Estimating corn leaf chlorophyll concentration from leaf and canopy reflectance. Remote Sens Environ.

[8] Haboudane D, Miller JR, Tremblay N, Zarco-Tejada PJ, Dextraze L (2002). Integrated narrow-band vegetation indices for prediction of crop chlorophyll content for application to precision agriculture. Remote Sens Environ.

[9] Dash J, Curran PJ (2004). The MERIS terrestrial chlorophyll index. Int J Remote Sens.

[10] Gitelson A, Kaufman YJ, Merzlyak MN (1996). Use of a green channel in remote sensing of global vegetation from EOS-MODIS. Remote Sens Environ.

[11] Gitelson A, Viña A, Ciganda V, Rundquist DC, Arkebauer TJ (2005). Remote estimation of canopy chlorophyll content in crops. Geophys Res Lett.

[12] Atzberger C (1995). The spectral correlation concept: An effective new image-based atmospheric correction methodology over land surfaces.

[13] Soenen SA, Peddle DR, Coburn CA, Hall RJ, Hall FG (2007). Canopy reflectance model inversion in multiple forward mode: Forest structural information retrieval from solution set distributions. Photogramm Eng Remote Sens.

[14] Meroni M, Colomgo R, Panigada C (2004). Inversion of a radiative transfer model with hyperspectral observations for LAI mapping in poplar plantations. Remote Sens Environ.

[15] Verhoef W (1984). Light scattering by leaf layers with application to canopy reflectance modeling: The SAIL model. Remote Sens Environ.

[16] Kimes DS, Knyazikhn Y, Privette JL, Abuelgasim AA, Gao F (2000). Inversion methods for physically-based models. Remote Sens Rev.

[17] Demarez V, Gastellu-Etchegorry J-P, Mougin E, Marty G, Proisy C, Dufrene E, Le Dantec V (1999). Seasonal variation of leaf chlorophyll content of a temperate forest. Inversion of the PROSPECT model. Int J Remote Sens.

[18] Ganapol BD, Johnson LF, Hammer PD, Hlavka CA, Peterson DL (1998). LEAFMOD: A new within-leaf radiative transfer model. Remote Sens Environ.

[19] Jacquemoud S, Baret F (1990). PROSPECT: A model of leaf optical properties spectra. Remote Sens Environ.

[20] Renzullo LJ, Blanchfield AL, Guillermin R, Powell KS, Held AA (2006). Comparison of PROSPECT and HPLC estimates of leaf chlorophyll contents in a grapevine stress study. Int J Remote Sens.

[21] Zarco-Tejada PJ, Miller JR, Noland TL, Mohammed GH, Sampson PH (2001). Scaling-up and model inversion methods with narrowband optical indices for chlorophyll content estimation in closed forest canopies with hyperspectral data. IEEE Trans Geosci Remote Sens.

[22] Datt B (1999). A new reflectance index for remote sensing of chlorophyll content in higher plants: Tests using eucalyptus leaves. J Plant Physiol.

[23] Pu R, Gong P, Yu Q (2008). Comparative analysis of EO-1 ALI and Hyperion, and Landsat ETM+ data for mapping forest crown closure and leaf area index. Sensors.

[24] Peddle DR, Johnson RL, Cihlar J, Leblanc SG, Chen JM, Hall FG (2007). Physically-based inversion modeling for unsupervised cluster labeling, independent forest classification and LAI estimation using MFM-5-scale. Can J Remote Sens.

[25] Gates DM (1965). Energy, plants, and ecology. Ecology.

[26] Knipling E (1970). Physical and physiological basis for the reflectance of visible and near-infrared radiation from vegetation. Remote Sens Environ.

[27] Curran PJ (1989). Remote sensing of foliar chemistry. Remote Sens Environ.

[28] Adam E, Mutunga O (2009). Spectral discrimination of papyrus vegetation (Cyperus papyrus L.) in swamp wetlands using field spectrometry. ISPRS J Photogramm Remote Sens.

[29] Asner GP (1998). Biophysical and biochemical sources of variability in canopy reflectance. Remote Sens Environ.

[30] van leeuwen WJD, Huete AR (1996). Effects of standing litter on the biophysical interpretation of plant canopies with spectral indices. Remote Sens Environ.

[31] Duncan J, Stow D, Franklin J, Hope A (1993). Assessing the relationship between spectral vegetation indices and shrub cover in the Jornada Basin, New Mexico. Int J Remote Sens.

[32] Graetz RD, Gentle MR (1982). The relationships between reflectance in the Landsat wavebands and the composition of an Australian semi-arid shrub rangeland. Photogramm Eng Remote Sens.

[33] Huete AR (1988). A soil-adjusted vegetation index (SAVI). Remote Sens Environ.

[34] Asrar G, Myneni R, Choudhury B (1992). Spatial heterogeneity in vegetation canopies and remote sensing of absorbed photosynthetically active radiation: A modeling study. Remote Sens Environ.

[35] Asner GP, Heidebrecht KB (2005). Desertification alters regional ecosystem climate interactions. Glob Change Biol.

[36] Yoder B, Pettigrew-Crosby RE (1995). Predicting nitrogen and chlorophyll content and concentrations from reflectance spectra (400–2500 nm) at leaf and canopy scales. Remote Sens. Environ.

[37] Zagolski F, Pinel V, Romier J, Alcayde D, Fontanari J, Gastellu-Etchegorry JP, Giordano G, Marty G, Mougin E, Joffre R (1996). Forest canopy chemistry with high spectral resolution remote sensing. Int J Remote Sens.

[38] Asner GP, Wessman CA, Archer S (1998a). Scale dependence of absorption of photosynthetically active radiation in terrestrial ecosystems. Ecol. Appl.

[39] Jago RA, Cutler MEJ, Curran PJ (1999). Estimating canopy chlorophyll concentration from field and airborne spectra. Remote Sens Environ.

[40] Daughtry C, Gallo K, Goward S, Prince S, Kustas W (1992). Spectral estimates of absorbed radiation and phytomass production in corn and soybean canopies. Remote Sens Environ.

[41] Jacquemoud S, Bacour C, Poilve H, Frangi J-P (2000). Comparison of four radiative transfer models to simulate plant canopies reflectance: direct and inverse mode. Remote Sens Environ.

[42] Gascon F, Gastellu-Etchegorry J-P, Lefevre-Fonollosa M-J, Dufrene E (2004). Retrieval of forest biophysical variables by inverting a 3-D radiative transfer model and using high and very high resolution imagery. Int J Remote Sens.

[43] Jacquemoud S (1993). Inversion of the PROSPECT + SAIL canopy reflectance model from AVIRIS equivalent spectra: Theoretical study. Remote Sens Environ.

[44] Chaurasia S, Dadhwal VK (2004). Comparison of principal component inversion with VI-empirical approach for LAI estimation using simulated reflectance data. Int J Remote Sens.

[45] Baret F, Hagolle O, Geiger B, Bicheron P, Miras B, Huc M, Berthelot B, Niño F, Weiss M, Samain O, Roujean JL, Leroy M (2007). LAI, fAPAR and fCover CYCLOPES global products derived from VEGETATION: Part 1: Principles of the algorithm. Remote Sens Environ.

[46] Houborg R, Boegh E (2008). Mapping leaf chlorophyll and leaf area index using inverse and forward canopy reflectance modeling and SPOT reflectance data. Remote Sens Environ.

[47] Gastellu-Etchegorry J-P, Demarez V, Pinel V, Zagolski F (1996a). Modeling radiative transfer in heterogeneous 3-D vegetation canopies. Remote Sens. Environ.

[48] Gastellu-Etchegorry J-P, Zagolski F, Romier J (1996b). A simple anisotropic reflectance model for homogeneous multilayer canopies. Remote Sens. Environ.

[49] Kimes DS, Kirchner JA (1983). Directional radiometric measurements of row-crop temperatures. Int J Remote Sens.

[50] Liang S (2004). Quantitative Remote Sensing of Land Surfaces.

[51] Kuusk A (1995a). A fast, invertible canopy reflectance model. Remote Sens. Environ.

[52] Moorthy I, Miller J, Noland T (2008). Estimating chlorophyll concentration in conifer needles with hyperspectral data: An assessment at the needle and canopy level. Remote Sens Environ.

[53] Chen JM, Leblanc SG (1997). A four-scale bi-directional reflectance model based on canopy architecture. IEEE Trans Geosci Remote Sens.

[54] Goel NS, Grier T (1988). Estimation of canopy parameters for inhomogeneous vegetation canopies from reflectance data: III. Trim: A model for radiative transfer in heterogeneous three-dimensional canopies. Remote Sens Environ.

[55] Huemmrich K (2001). The GeoSail model: A simple addition to the SAIL model to describe discontinuous canopy reflectance. Remote Sens Environ.

[56] Schlerf M, Atzberger C (2006). Inversion of a forest reflectance model to estimate structural canopy variables from hyperspectral remote sensing data. Remote Sens Environ.

[57] Goel NS, Thompson RL (2000). A snapshot of canopy reflectance models and a universal model for the radiation regime. Remote Sens Rev.

[58] Govaerts YM, Verstraete MM (1998). Raytran: A Monte Carlo ray-tracing model to compute light scattering in three-dimensional heterogeneous media. IEEE Trans Geosci Remote Sens.

[59] Borel C, Gerstl S, Powers B (1991). The radiosity method in optical remote sensing of structured 3-D surfaces. Remote Sens Environ.

[60] Borel CC, Gerstl SAW (1992). Adjacency-blurring-effect of scenes modeled by the radiosity method. Atmos Propag Remote Sens.

[61] Chelle M, Andrieu B (1998). The nested radiosity model for the distribution of light within plant canopies. Ecol Model.

[62] Kimes DS, Nelson RF, Manry MT, Fung AK (1998). Review article: Attributes of neural networks for extracting continuous vegetation variables from optical and radar measurements. Int J Remote Sens.

[63] Atzberger C (1997). Estimates of Winter Wheat Production through Remote Sensing and Crop Growth Modeling.

[64] Bicheron P, Leroy M (1999). A method of biophysical parameter retrieval at global scale by inversion of a vegetation reflectance model. Remote Sens Environ.

[65] Jacquemoud S, Baret F, Andrieu B, Danson FM, Jaggard K (1995). Extraction of vegetation biophysical parameters by inversion of the PROSPECT + SAIL models on sugar beet canopy reflectance data: Application to TM and AVIRIS sensors. Remote Sens Environ.

[66] Combal B, Baret F, Weiss M, Trubuil A, Mace D, Pragnere A, Myneni R, Knyazikhin Y, Wang L (2003). Retrieval of canopy biophysical variables from bidirectional reflectance Using prior information to solve the ill-posed inverse problem. Remote Sens Environ.

[67] Combal B, Baret F, Weiss M (2002). Improving canopy variables estimation from remote sensing data by exploiting ancillary information: Case study on sugar beet canopies. Agronomie.

[68] Gastellu-Etchegorry J-P, Gascon F, Esteve P (2003). An interpolation procedure for generalizing a look-up table inversion method. Remote Sens Environ.

[69] Weiss M, Baret F, Myneni RB, Pragnere A, Knyazikhin Y (2000). Investigation of a model inversion technique to estimate canopy biophysical variables from spectral and directional reflectance data. Agronomie.

[70] Fang H, Liang D (2005). A hybrid inversion method for mapping leaf area index from MODIS data: Experiments and application to broadleaf and needleleaf canopies. Remote Sens Environ.

[71] Gopal S, Woodcock C (1996). Remote sensing of forest change using artificial neural networks. IEEE Trans Geosci Remote Sens.

[72] Walthall C, Dulaney W, Anderson M, Norman J, Fang H, Liang S (2004). A comparison of empirical and neural network approaches for estimating corn and soybean leaf area index from Landsat ETM+ imagery*1. Remote Sens Environ.

[73] Weiss M, Baret F (1999). Evaluation of canopy biophysical variable retrieval performances from the accumulation of large swath satellite data. Remote Sens Environ.

[74] Durbha S, King R, Younan N (2007). Support vector machines regression for retrieval of leaf area index from multiangle imaging spectroradiometer. Remote Sens Environ.

[75] Atzberger C (2004). Object-based retrieval of biophysical canopy variables using artificial neural nets and radiative transfer models. Remote Sens Environ.

[76] Atzberger C, Jarmer T, Schlerf M, Kötz B, Werner W, Habermeyer M, Müller A, Holzwarth S (2003). Retrieval of wheat bio-physical attributes from hyperspectral data and SAILH+PROSPECT radiative transfer mode.

[77] Danson FM, Rowland CS, Baret F (2003). Training a neural network with a canopy reflectance model to estimate crop leaf area index. Int J Remote Sens.

[78] Zarco-Tejada P, Miller JR, Morales A, Berjon A, Aguera J (2004b). Hyperspectral indices and model simulation for chlorophyll estimation in open-canopy tree crops. Remote Sens. Environ.

[79] Disney M, Lewis P, Saich P (2006). 3D modelling of forest canopy structure for remote sensing simulations in the optical and microwave domains. Remote Sens Environ.

[80] Eklundh L, Harrie L, Kuusk A (2001). Investigating relationships between Landsat ETM+ sensor data and leaf area index in a boreal conifer forest. Remote Sens Environ.

[81] Fang H, Liang S, Kuusk A (2003). Retrieving leaf area index using a genetic algorithm with a canopy radiative transfer model. Remote Sens Environ.

[82] Fernandes R, Butson C, Leblanc S, Latifovic R (2003). Landsat-5 TM and Landsat-7 ETM + based accuracy assessment of leaf area index products for Canada derived from SPOT-4 VEGETATION data. Can J Remote Sens.

[83] Gemmell F, Varjo J, Strandstrom M, Kuusk A (2002). Comparison of measured boreal forest characteristics with estimates from TM data and limited ancillary information using reflectance model inversion. Remote Sens Environ.

[84] Kotz B, Schaepman M, Morsdorf F, Bowyer P, Itten K, Allgower B (2004). Radiative transfer modeling within a heterogeneous canopy for estimation of forest fire fuel properties. Remote Sens Environ.

[85] Zarco-Tejada P, Miller JR, Harron J, Hu B, Noland TL, Goel N, Mohammed GH, Sampson P (2004a). Needle chlorophyll content estimation through model inversion using hyperspectral data from boreal conifer forest canopies. Remote Sens. Environ..

[86] Allen WA, Richardson AJ (1968). Interaction of light with a plant canopy. J Opt Soci Amer.

[87] Zhang N, Zhao Y (2009). Estimating leaf area index by inversion of reflectance model for semiarid natural grasslands. Sci China D Earth Sci.

[88] Pragnère A, Baret F, Weiss M, Myneni R, Knyazikhin Y, Wang L (1999). Comparison of three radiative transfer model inversion techniques to estimate canopy biophysical variables from remote sensing data.

[89] CROMA (2000). Crop reflectance operational models for agriculture. Description of Energy, Environment and Sustainable Development Work Programme.

[90] He Y, Guo X, Si BC (2007). Detecting grassland spatial variation by a wavelet approach. Int J Remote Sens.

[91] Bacour C, Jacquemoud S, Tourbier Y, Dechambre M, Frangi J (2002). Design and analysis of numerical experiments to compare four canopy reflectance models. Remote Sens Environ.

